# Reproducibility of Facial Information in Three-Dimensional Reconstructed Head Images: An Exploratory Study

**DOI:** 10.2174/1573405619666230123105057

**Published:** 2023-06-02

**Authors:** Tatsuya Uchida, Taichi Kin, Katsuya Sato, Tsukasa Koike, Satoshi Kiyofuji, Yasuhiro Takeda, Ryoko Niwa, Toki Saito, Ikumi Takashima, Takuya Kawahara, Satoru Miyawaki, Hiroshi Oyama, Nobuhito Saito

**Affiliations:** 1 Department of Neurosurgery, The University of Tokyo, 7-3-1 Hongo, Bunkyo-ku, Tokyo 113-8655, Japan;; 2 Department of Clinical Information Engineering, Graduate School of Medicine, The University of Tokyo, 7-3-1 Hongo, Bunkyo-ku, Tokyo 113-8655, Japan;; 3 Data Science Office, Clinical Research Promotion Center, The University of Tokyo Hospital, 7-3-1 Hongo, Bunkyo-ku, Tokyo 113-8655, Japan

**Keywords:** Biomedical imaging, computed tomography, face recognition, personally identifiable information, three-dimensional reconstruction, face detection

## Abstract

**Background:**

Facial information acquired *via* three-dimensional reconstruction of head computed tomography (CT) data may be considered personal information, which can be problematic for neuroimaging studies. However, no study has verified the relationship between slice thickness and face reproducibility. This study determined the relationship and match rate between image slice thickness and face detection accuracy of face-recognition software in facial reconstructed models.

**Methods:**

Head CT data of 60 cases comprising entire faces obtained under conditions of non-contrast and 1-mm slice thickness were resampled to obtain 2-10-mm slice-thickness data. Facial models, reconstructed by image thresholding, were acquired from the data. We performed face detection tests per slice thickness on the models and calculated the face detection rate. The reconstructed facial models created from 1-mm slice-thickness data and other slice thicknesses were used as training and test data, respectively. Match confidence scores were obtained *via* three programs, match rates were calculated per slice thickness, and generalized estimating equations were used to evaluate the match rate trend.

**Results:**

In general, the face detection rates for the 1-10-mm slice thicknesses were 100, 100, 98.3, 98.3, 95.0, 91.7, 86.7, 78.3, 68.3, and 61.7%, respectively. The match rates for the 2-10-mm slice thicknesses were 100, 98.3, 98.3, 95.0, 85.0, 71.7, 53.3, 28.3, and 16.7%, respectively.

**Conclusion:**

The reconstructed models tended to have higher match rates as the slice thickness decreased. Thus, thin-slice head CT imaging data may increase the possibility of the information becoming personally identifiable health information.

## 1 INTRODUCTION

In the medical field, data sharing to promote research activities is widely encouraged; however, consideration for personal information protection is becoming stronger [1, 2]. It has been reported that head medical image data, including those of the facial region, can generate facial contours under three-dimensional (3D) reconstruction, which may be used to identify the patient [3, 4]. Schwarz *et al*. reported that using automatic facial recognition software, they could correctly match a person's face photo with the 3D computer graphics model created from 3D fluid-attenuated inversion recovery head magnetic resonance imaging (MRI) with 1.2-mm slice thicknesses at a rate of 83% [5].

Facial reconstructed models are not explicitly mentioned in the Health Insurance Portability and Accountability Act in the United States or the General Data Protection Regulation in Europe; however, there are concerns that they may fall under the category of personally identifiable images [6]. The Act on Anonymized Medical Data That Are Meant to Contribute to Research and Development in the Medical Field in Japan clearly states that head medical image data must be treated as personal information depending on the reproducibility of the 3D reconstruction. Although the accuracy of 3D contouring from extant medical images is affected by slice thickness [7, 8], no report has verified the relationship between slice thickness and the reproducibility of facial reconstructed models. Such an evaluation may allow for the estimation of the borderline slice thicknesses that should be treated as personal information, which is important in neuroimaging studies.

Studies have used software-based facial recognition matches as an indicator to determine personal information that constitutes a facial image [5, 9]. In this study, the relationship between the slice thickness of the original image and the accuracy of face detection using face-recognition software was evaluated in reconstructed facial models created by 3D reconstruction from head computed tomography (CT). Then, the match rate of facial information in the facial reconstructed models created for each slice thickness was evaluated and the relationship between slice thickness and face reproducibility was determined.

## 2 MATERIALS AND METHODS

### 2.1 Image Datasets

**Fig. (1):**
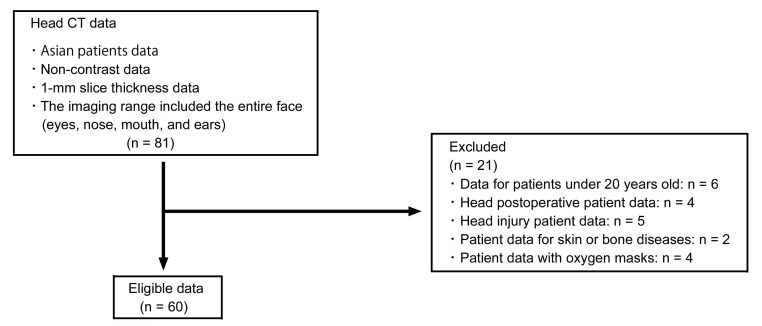
Flow diagram of the study. CT = computed tomography.

Non-contrast head CT data from 60 Asian patients (24 men and 36 women, age 21−92 years) without facial skin diseases, and taken between January 2017 and July 2021, were included in this study (Table **1**). Data from head injury patients, patients after head surgery, and patients wearing oxygen masks were excluded (Fig. **1**). CT scans were performed using a 320-row multi-slice CT scanner (Aquilion ONE; Toshiba Medical Systems, Tokyo, Japan). The CT imaging parameters were as follows: collimation, 0.5 mm; tube voltage, 120 kV; tube current, 200 mA; rotation time, 0.6 s; reconstruction section width, 0.5 mm; reconstruction interval, 0.5 mm; and voxel size, 0.43 × 0.43 × 1.00 mm. The imaging range included the entire face (*i.e*., eyes, nose, mouth, and ears) (Fig. **1**). The internal review board of the University of Tokyo Hospital approved the study protocol (consent number 2021107NI) and written informed consent was obtained from all patients before their participation.

### 2.2 Methods for Creating Facial Reconstructed Models

**Fig. (2):**
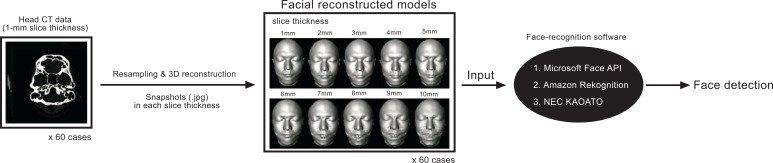
Procedure for face detection test. Using image processing software, head CT data with 1-mm slice thickness were resampled to create reconstructed models of 10 slice thicknesses, and snapshots (.jpg) of these models were acquired. These snapshots (for 60 cases) were input into the face-recognition software, and face detection tests were performed. CT = computed tomography; 3D = three-dimensional.

CT data of sixty cases were input into the imaging software, Amira^®^ (version 2019.4; Thermo Fisher Scientific, Waltham, USA), in the Digital Imaging and Communications in Medicine format. CT data with a 1-mm slice thickness was resampled using the Lanczos method (which computes new values for the sampled data *via* interpolation) [10], and CT data with 2-10-mm slice thicknesses (1-mm increments) were acquired without changing the resolution of the fault plane (X and Y directions). The 3D reconstruction was performed using image thresholding with an optimal threshold (-200 HU) for a face contouring display by surface rendering, and reconstructed facial models with slice thicknesses of 1, 2, 3, 4, 5, 6, 7, 8, 9, and 10 mm were created (Fig. **2**). The human body below the mandible and the head fixture was removed. The reconstructed models were gray, and directional light was used.

### 2.3 Face Detection Test

For each slice-thickness-based reconstructed facial model, the frontal position was determined by the author, and snapshots (.jpg) were taken (60 cases, 600 snapshots). These snapshots were input into three automatic face-recognition software packages: Face API (Microsoft Corp., Washington, USA), Rekognition (Amazon.com, Inc., Washington, USA), and KAOATO (NEC Corp., Tokyo, Japan), and face detection test was performed (Fig. **2**). The test identifies facial landmarks, detects human faces in the input image, and returns the coordinates of the rectangle at that position. The face detection rate for each slice thickness and 95% confidence interval (CI) were calculated for each of the three face-recognition programs.


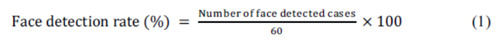


### 2.4 Face-recognition Methods

Using the same software, face-recognition tests (verification of the same person) were performed using the reconstructed facial models of the slice thickness with the highest face detection rate and the thinnest slice thickness as the gold standard (training data) and the facial reconstructed models of other slice thicknesses as the test data.

#### 2.4.1 Training Data

In the reconstructed facial models with the highest face detection rate and the thinnest slice thickness, seven snapshots (.jpg) were taken for each person at different angles: straight ahead, 10° and 20° left and right, and 10° up and down (60 cases, 420 snapshots). These snapshots were used as training data for the automatic face-recognition software.

#### 2.4.2 Test Data

Snapshots of reconstructed facial models with nine slice thicknesses other than the one used in the training data (straight ahead only, 60 cases, 540 snapshots) were used as test data for the automatic face-recognition software.

#### 2.4.3 Face-Recognition Test

The test data were input into the face-recognition software, and the match confidence score with the training data was output. The match confidence score of Face API and Rekognition (software) ranged from 0 to 1, where “1” is a perfect match, and “0” is a complete mismatch. The KAOATO (software) match confidence score ranged from 0 to 100, where “100” is a perfect match, and “0” is a complete mismatch. The detailed methodology of face recognition is an undisclosed technology unique to each software. A “match” is defined as when the training data has the highest match confidence score and the input test data display the same person. The match rate for each slice thickness and 95% CI were calculated for each of the three face-recognition programs.


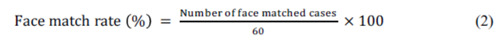


### 2.5 Analysis Methods

Generalized estimating equations were used to evaluate the trend of the match rate with slice thickness, and the odds ratio and 95% CI were calculated using “match” or “no match” (binary variable) as the objective variable and “slice thickness” (continuous variables: nine slice thicknesses other than the one used in the training data) as the dependent variable. Statistical analyses were performed using SAS version 9.4 (SAS Institute Inc.).

## 3 RESULTS

The reconstructed facial models of each slice thickness were created for all CT data, face detection tests were performed, and face recognition tests were performed using facial recognition software.

**Fig. (3):**
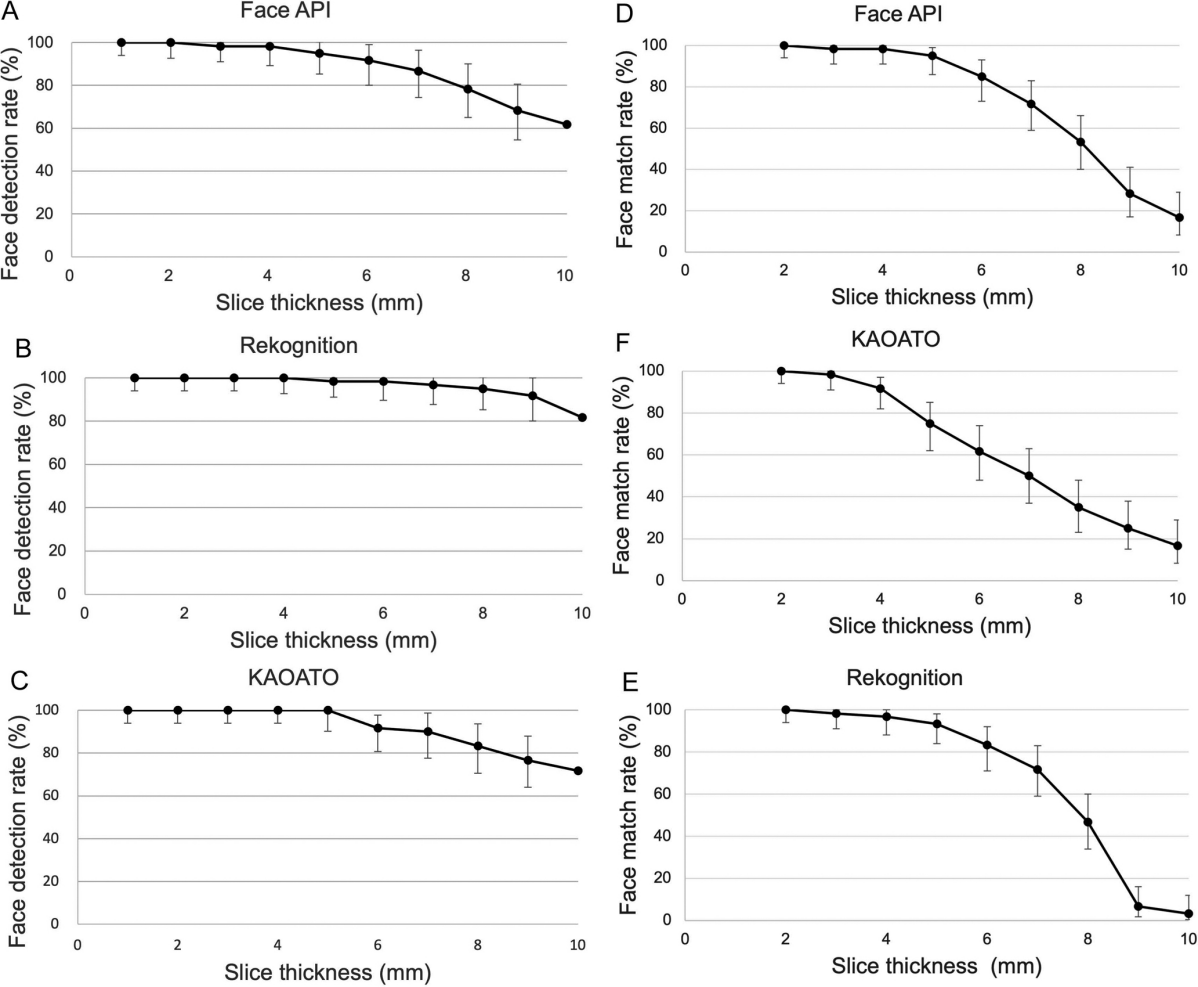
Face detection and match rates of test data for each slice thickness in three face-recognition software packages. The Clopper-Pearson exact 95% confidence interval was used. (**A**): Relationship between slice thickness and face detection rate in Microsoft Face API. (**B**): Relationship between slice thickness and face detection rate in Amazon Rekognition. (**C**): Relationship between slice thickness and face detection rate in NEC KAOATO. (**D**): Relationship between slice thickness and match rate in Microsoft Face API. (**E**): Relationship between slice thickness and match rate in Amazon Rekognition. (**F**): Relationship between slice thickness and match rate in NEC KAOATO.

The Face API output face detection rate of 100% (60/60, CI 95%: 94−100% according to the Clopper-Pearson exact method), 100% (60/60, 94−100%), 98.3% (59/60, 91−100%), 98.3% (59/50, 91−100%), 95.0% (57/60, 86−99%), 91.7% (55/60, 82−97%), 86.7% (52/60, 75−94%), 78.3% (47/60, 66−88%), 68.3% (41/60, 55−80%), and 61.7% (37/60, 48−74%) for slice thicknesses of 1, 2, 3, 4, 5, 6, 7, 8, 9, and 10 mm, respectively (Fig. **3A**).

The same trend was observed for Rekognition (Fig. **3B**) and KAOATO (Fig. **3C**). Face detection was successful in all cases with 1- and 2-mm slice thicknesses for the three software. The median thickest slice for successful face detection was 10.0 mm for all tools.

**Fig. (4):**
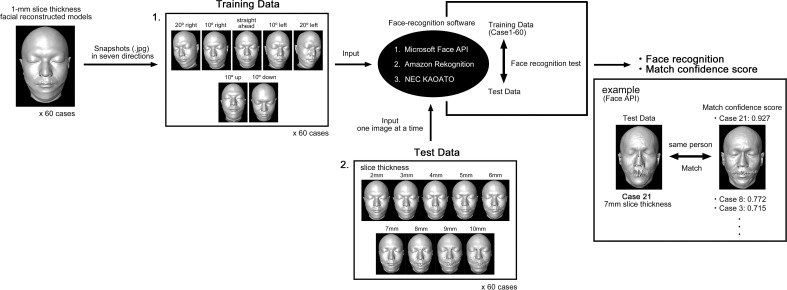
Procedure for face-recognition test. Snapshots (.jpg) in seven directions of 1-mm slice-thickness reconstructed facial models were input into the face-recognition software as training data. Snapshots (for 60 cases) of reconstructed facial models with 2-10-mm slice thicknesses (in increments of 1 mm) were used as test data, and each snapshot was input into the face-recognition software for face recognition. The input test and training data were tested for face recognition, and the match confidence scores were output. A “match” was defined when the training data with the highest match confidence score and the input test data displayed the same person. The example shows the input of test data for Case 21 with a slice thickness of 7 mm. With Face API, the 7-mm slice-thickness reconstructed facial model for Case 21 was matched. The match confidence score was 0.927. Numbers 1 and 2 are the sequence to be input into the facial recognition software.

For the face-recognition test, the reconstructed facial models with 1-mm slice thickness were used as training data, and the reconstructed facial models with 2-10-mm slice thicknesses (in increments of 1 mm) were used as test data for face-recognition (Fig. **4**).

The match rate for Face API, was 100% (60/60, CI 95%: 94−100% by the Clopper-Pearson exact method), 98.3% (59/60, 91−100%), 98.3% (59/50, 91−100%), 95.0% (57/60, 86−99%), 85.0% (51/60, 73−93%), 71.7% (43/60, 59−83%), 53.3% (32/60, 40−66%), 28.3% (17/60, 17−41%), and 16.7% (10/60, 8−29%) for 2, 3, 4, 5, 6, 7, 8, 9, and 10 mm, respectively (Fig. **3D**).

The same trend was observed for Rekognition (Fig. **3E**) and KAOATO (Fig. **3F**). The median thickness of the thickest slice for a successful match was 8.0 mm for Face API, 7.0 mm for Rekognition, and 6.5 mm for KAOATO.

Using generalized estimating equations, the match rate tended to decrease with increasing slice thickness in all software (Face API; OR: 0.410, 95% CI: 0.334−0.503, P < 0.0001, Rekognition; OR: 0.306, 95% CI: 0.221−0.423, P < 0.0001, KAOATO; OR: 0.517, 95% CI: 0.453−0.590, P < 0.0001).

Fig. (**5**) shows a case (Case 21) of a 36-year-old Japanese man. Face API and KAOATO successfully detected the face even with a 10-mm slice thickness. Rekognition could not detect faces using a 10-mm slice thickness. Face API and KAOATO matched faces up to 8-mm slice thickness, and Rekognition matched faces up to 7-mm slice thickness. The reconstructed facial models with 9- and 10-mm slice thicknesses did not display a match using Face API, and the match confidence score was the highest in Case 8.

## 4 DISCUSSION

Using the head CT data, the face detection and match rates were analyzed for each slice thickness, and the trend of the match rate with slice thickness was evaluated to verify the relationship between slice thickness and face reproducibility. For all software, the face detection and match rates for data obtained from the 2-mm-thick slices data were 100%. Additionally, the greater the CT slice thickness, the lower the match rate. The match rate was less than 60% for slice thicknesses of at least 8 mm.

### 4.1 Anonymity of Facial Reconstructed Models

Several reports have verified the possibility of identifying individuals from facial models reconstructed using CT and MRI. Human visual evaluation has shown that the identification rate of reconstructed facial models is approximately 40-61% [3, 4], and that reconstructed facial models are poor substitutes for photographs, although they pose a potential privacy risk [11]; however, a recent study reported that 83% of reconstructed facial models could be personally identified using highly accurate facial recognition software powered by artificial intelligence [5].

In this study, tested data with a maximum slice thickness of 4 mm showed a match rate of more than 80%. Because it was only face-recognition tests with training data based on reconstructed models, careful interpretation is needed to determine whether individuals can be identified. The anonymity of face images, not only medical images, is not well defined and is difficult to interpret from calculated values [12].

Facial recognition software is evolving at a rapid pace, and the difficulty in protecting personal information will increase in the future. Research is needed to develop optimal and improved anonymization methods for medical images of the head and face.

### 4.2 Relationship Between Slice Thickness and Face Reproducibility

In CT images, it is known that the greater the slice thickness, the lower the accuracy of contouring [13-15]. At greater slice thicknesses, the contouring accuracy of the reconstructed facial models decreases, which reduces reproducibility, possibly the reason for the reduced match rate with greater slice thicknesses in this study, for which reproducibility was low.

Previous reports on image resolution and personal identification have also shown that the personal identification rate decreases with the resolution [16]. For this study, greater slice thicknesses may have reduced the resolution between faults, resulting in a lower match rate. This is the first study that focuses on slice thickness and face reproducibility.

### 4.3 Clinical Application

In clinical practice, head CT is usually performed with a 3−5-mm slice thickness; however, the recommended CT slice thickness could vary depending on the disease (reason for CT examination) [17]. In this study, both face detection and match rates were 100% for the 2-mm slice-thickness data, suggesting the necessity of emphasizing the personal information in the image data in cases where thin-slice head CT imaging data are recommended, such as in facial trauma cases [18].

In general, the match rate of less than 60% (for slice thicknesses of at least 8 mm in this study) is likely to make identifying individuals difficult [3, 4]. When sharing head medical image data, 2-mm slice-thickness data require anonymizing, while 8-mm slice-thickness data (the match rate in this study was less than 60%) may not require anonymizing in many cases.

This study recognized a difference in the anonymity of the image data because the change in the match rate depends on the slice thickness, which is important in personal information protection.

### 4.4 Limitations and Future Work

This study verified the relationship between slice thickness and face reproducibility and did not propose a specific anonymization method. In the future, it will be necessary to develop a new anonymization method based on the results of this exploratory study. This study, which showed how much slice thickness is a high risk for face identification, can help in the development of anonymization methods.

The presence or absence of tooth artifacts may have affected face detection and match rates. Only three face-recognition programs were used in this study, and the results may change significantly with software developments in the future.

As this study was conducted on a small scale (60 cases), the face detection and match rate could be improved with a large amount of training data. In addition, the training and test data both used snapshots of reconstructed models, which may overestimate the match rate.

In this study, data including the entire face (*i.e*., eyes, nose, mouth, and ears) were used; however, the range of imaging in clinical settings varies and the relationship between the range of imaging and face detection/recognition needs to be investigated.

## CONCLUSION

In the reconstructed facial models created from head CT data, the face detection and match rates per slice thickness were quantitatively analyzed, and the relationship between the slice thickness and reproducibility of the l reconstructed facia models was evaluated. The greater the slice thickness, the lower the match rate with the training data, with the match rate less than 60% for slice thicknesses of at least 8 mm. The face detection and match rates for data obtained from the 2-mm-thick slice-thickness data were both 100%. Thus, thin-slice head CT imaging data may increase the possibility of the information becoming personally identifiable health information.

## Figures and Tables

**Fig. (5):**
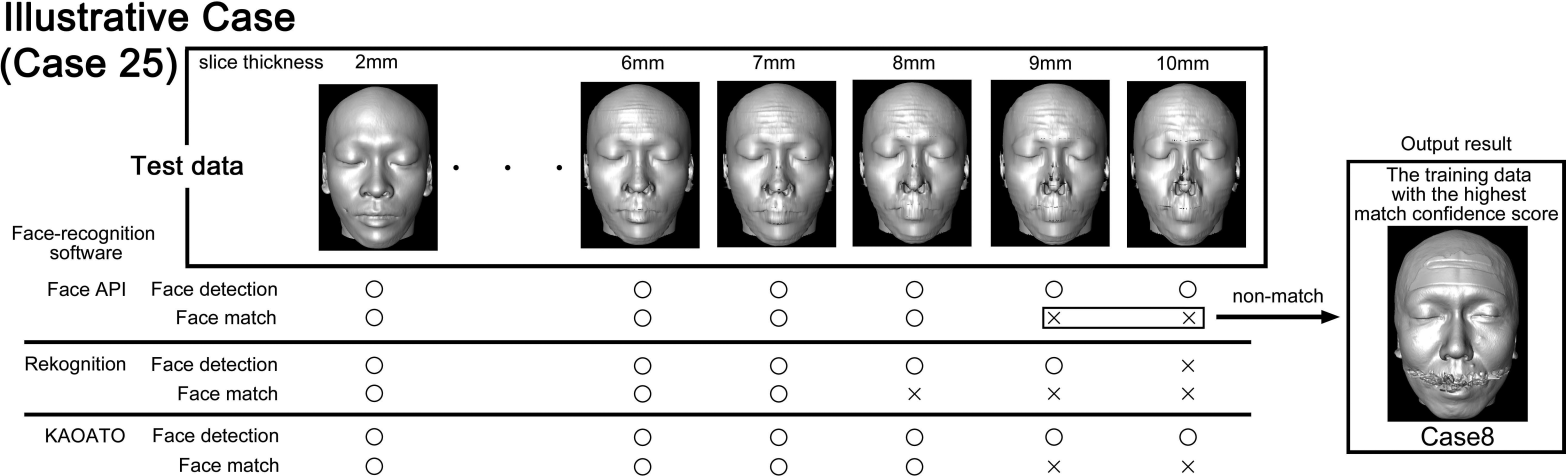
Illustrative case (Case 25). This is a case of a 36-year-old Japanese man. Face detection was successful in Face API and KAOATO, even with a slice thickness of 10 mm. In Rekognition, no face was detected with a 10-mm slice thickness. Face API, Rekognition, and KAOATO matched faces up to 8-, 7-, and 8-mm slice thicknesses. With Face API, the facial reconstructed models with 9- and 10-mm slice thicknesses did not match, and the match confidence score was the highest in Case 8. Successful face detection is marked with “O” and unsuccessful face detection is marked with “×.” Successful face match is marked as “O” and unsuccessful face match is marked as “×”.

**Table 1:** Characteristics of participant data.

**Characteristics**	**Value**
No. of participants Age (y)* Sex	60 53 ± 17 [21-92]
Female	24 (40)
Male	36 (60)
Diagnosis	
Cerebrovascular disease	10 (17)
Brain tumor	33 (55)
Functional disorder	13 (22)
Others	4 (7)

**Note:** Except where indicated, data are numbers of patients, with percentages in parentheses.

*Age is expressed in years as the mean ± standard deviation, with the range in brackets.

## Data Availability

Data supporting the results of this study are available upon request from the corresponding author. However, access to these data is limited, and the data will not be made available to the public. The reasons for this are listed below. Although this study was conducted with the written consent of the participants, each image contains facial information and requires separate additional consent from each participant to be used as a figure or made available to the public. Our goal was to develop an anonymization method for medical images. This study was an exploratory study for this purpose, and we will use the data from this study for future research.

## LIST OF ABBREVIATIONS

3D: Three-Dimensional
CI: Confidence Interval
CT: Computed Tomography
MRI: Magnetic Resonance Imaging
